# Higher Prevalence of the Periodontal Pathogen *Selenomonas noxia* among Pediatric and Adult Patients May Be Associated with Overweight and Obesity

**DOI:** 10.3390/pathogens13040338

**Published:** 2024-04-19

**Authors:** Austin Williams, Jace Porter, Karl Kingsley, Katherine M. Howard

**Affiliations:** 1 Department of Advanced Education in Pediatric Dentistry, School of Dental Medicine, University of Nevada, Las Vegas, 1700 West Charleston Blvd, Las Vegas, NV 89106, USA; 2 Department of Clinical Sciences, School of Dental Medicine, University of Nevada, Las Vegas, 1700 West Charleston Blvd, Las Vegas, NV 89106, USA; 3 Department of Biomedical Sciences, School of Dental Medicine, University of Nevada, Las Vegas, 1001 Shadow Lane, Las Vegas, NV 89106, USA; katherine.howard@unlv.edu

**Keywords:** *Selenomonas noxia*, overweight, obese, saliva screening, qPCR, prevalence

## Abstract

New evidence has suggested that oral and gut microflora may have significant impacts on the predisposition, development, and stability of obesity in adults over time—although less is known about this phenomenon in children. Compared with healthy-weight controls, overweight and obese adult patients are now known to harbor specific pathogens, such as *Selenomonas noxia* (*S. noxia*), that are capable of digesting normally non-digestible cellulose and fibers that significantly increase caloric extraction from normal dietary intake. To evaluate this phenomenon, clinical saliva samples (N = 122) from subjects with a normal BMI (18–25) and a BMI over 25 (overweight, obese) from an existing biorepository were screened using qPCR. The prevalence of *S. noxia* in samples from normal-BMI participants were lower (21.4%) than in overweight-BMI (25–29; 46.1%) and obese-BMI (30 and above; 36.8%) samples—a strong, positive correlation that was not significantly affected by age or race and ethnicity. These data strongly suggest that *S. noxia* may be intricately associated with overweight and obesity among patients, and more research will be needed to determine the positive and negative feedback mechanisms that may be responsible for these observations as well as the interventions needed to remove or reduce the potential effects of this oral pathogen.

## 1. Introduction

The oral microbiome may exhibit many significant effects on different aspects of oral and systemic health in humans [1,2]. Several systematic reviews have demonstrated, for example, the clinical efficacy of oral and gastrointestinal microbiome therapies that have significantly improved patient symptoms and outcomes in autoimmune, rheumatoid, and hypersensitivity diseases, such as atopic eczema [3,4]. Moreover, strong evidence has developed that specifically implicates the oral microbiome in systemic inflammation and the interrelated connections with overall patient health and disease [5,6].

Recent evidence now suggests that oral and gut microflora may have significant impacts on the predisposition, development, and stability of obesity in adults over time [7,8]. Most of the focus of these investigations has been on the two-way relationships between the oral microbiome and periodontal disease with inflammation, cardiovascular disease, and obesity [9,10,11]. Although the pathogens involved in periodontal disease are mainly found in older adults, evidence has emerged that demonstrates some of these pathogens may also be found in children—and may be associated with the development and progression of childhood obesity [12,13,14,15]. 

More specifically, systematic reviews of oral and gut microbiome studies have found that the lack of specific constituents, such as *Akkermansia muciniphila*, may be strongly associated with overweight and obesity among patients [16,17]. Moreover, studies involving the supplementation of *Akkermansia* have demonstrated clinical efficacy in modulating or mediating obesity in some patients [18,19]. Whether there are additional oral and gastrointestinal microbes that might be responsible for outcompeting *Akkermansia* and shifting the microbial composition towards obesity is not yet well understood [20,21]. 

However, recent evidence has now suggested that a particular periodontal pathogen, *Selenomonas noxia*, may be capable of digesting cellulose and other normally non-digestible fibers that significantly increase calorie extraction from normal dietary intake compared with healthy-weight adults who do not harbor this pathogen [22,23]. Although *Selenomonas* has been identified in periodontal disease and obesity studies in adults, more recent studies from this institution have demonstrated that this organism may also be present in saliva from a significant percentage of pediatric patients [24,25,26]. These observations suggest that *Selenomonas* may be systemically present in pediatric patients and may influence the development and progression of obesity over time—although no comparative studies of *Selenomonas* among adult and pediatric overweight and obese patients has yet been conducted. 

Based upon this lack of information, the primary goal and research question of this study was to evaluate salivary samples for the presence of *Selenomonas* from overweight and obese patients for comparison to samples from patients with normal body mass index (BMI) in both children and adults.

## 2. Materials and Methods

### 2.1. Study Approval

This study was conducted according to the Declaration of Helsinki, and the protocol for this study was reviewed and approved by the Office for the Protection of Research Subjects (OPRS) and the Institutional Review Board (IRB) at the University of Nevada, Las Vegas. The protocol “Retrospective analysis of microbial prevalence from DNA isolated from saliva samples originally obtained from the University of Nevada, Las Vegas (UNLV) School of Dental Medicine (SDM) pediatric and clinical population” was deemed research exempt, as this study is a retrospective investigation of samples within an existing biorepository and does not involve interactions with patients, the collection of patient samples or information, and does not contain any patient identifying, sensitive personal, or health information that could be linked with any current or former patient. These guidelines match the Department of Health and Human Services (HHSs) regulations for 45 CFR 46, which involve exempt research.

### 2.2. Original Sample Collection

The DNA samples used in the current study were isolated from saliva that was originally obtained from clinic patients at the UNLV-SDM pediatric and adult dental care clinics. The protocol for these collections was also reviewed by the OPRS and approved by the IRB under “The Prevalence of Oral Microbes in Saliva from the UNLV School of Dental Medicine Pediatric and Adult Clinical Population”. In brief, all the adult study participants were volunteers and had to provide informed consent. In addition, pediatric study participants were also voluntary and had to provide pediatric assent, as well as the informed consent of the child’s parent or guardian. Inclusion criteria included patients of record at UNLV-SDM and voluntary participation. Exclusion criteria included any individuals who were not a patient of record at UNLV-SDM, did not have demographic information or BMI data or calculations, any person who declined to participate, and any person that was not willing or able to provide informed consent or pediatric assent, as designated.

Saliva samples were collected in sterile 50 mL collection tubes and brought to a Biosafety Level 2 (BSL-2) laboratory for storage, subsequent processing, and analysis. Limited demographic data were collected for each patient, which consisted solely of sex, age at the time of collection, race or ethnicity (self declared), and body mass index or BMI calculated using the recorded height and weight of the patient at the time of sample collection. To maintain anonymity, all samples were labeled with randomly generated, non-duplicated identifiers that could not be linked with any patient information.

### 2.3. DNA Isolation

The samples were then processed to isolate DNA, as previously described [24,25,26]. In brief, the TRizol reagent from Fisher Scientific (Fair Lawn, NJ, USA) was used to perform DNA extractions from each sample according to the manufacturer’s recommendations. Following the addition of chloroform and centrifugation, the upper phase was transferred to a new sterile microcentrifuge tube and isopropanol was added to precipitate the DNA. Following centrifugation, the DNA-containing pellet was washed with ethanol and re-centrifuged. The pellet was then resuspended in 100 uL of nuclease-free water for analysis and screening. The analysis of the DNA isolated from each sample was performed by using the NanoDrop 2000 spectrophotometer system from Fisher Scientific (Fair Lawn, NJ, USA), which measures the absorbance readings of liquid samples at A260 and A280 nm to provide qualitative and quantitative estimations.

### 2.4. qPCR Screening

The screening of samples was performed using the SYBR green master mix obtained from Fisher Scientific (Fair Lawn, NJ, USA). All samples were screened for the presence of human DNA using the positive control primers for glyceraldehyde 3-phosphate dehydrogenase or GAPDH (metabolic standard) and beta actin (structural standard). In addition, all the samples were subsequently screened for the presence of bacterial DNA using the positive control primer for 16S rRNA. All the samples that tested positive for both human and bacterial DNA (as contained in saliva samples) were then screened for the presence of *Selenomonas* noxia or *S. noxia*. All reactions were performed using the QuantStudio quantitative polymerase chain reaction (PCR) system from Applied Biosystems (Waltham, MA, USA) and primers synthesized from Eurofins Scientific (Louisville, KY, USA), as follows:

*Positive controls*

(Human) GAPDH forward, 5′ATCTTCCAGGAGCGAGATCC-3′(Human) GAPDH reverse, 5′ACCACTGACACGTTGGCAGT-3′(Human) Beta actin forward, 5′-GTGGGGTCCTGTGGTGTG-3′(Human) Beta actin reverse, 5′-GAAGGGGACAGGCAGTGA-3′(Bacterial) 16S rRNA forward, 5′-ACGCGTCGACAGAGTTTGATCCTGGCT-3′(Bacterial) 16S rRNA reverse, 5′-GGGACTACCAGGGTATCTAAT-3′

*Selenomonas noxia*

SN forward primer:5′-TCTGGGCTACACACGTACTACAATG-3′SN reverse primer: 5′-GCCTGCAATCCGAACTGAGA-3′

### 2.5. Statistical Analysis

Demographic variables and characteristics, such as sex, age and race or ethnicity, were compiled and summarized with descriptive statistics, such as percentages, using Microsoft Excel 2021, Office 365 Version from Microsoft (Redmond, Washington, DC, USA). Differences between parametric data within the study sample and the overall clinic population, such as patient age, were calculated using two-tailed Student’s *t*-tests. Differences between the percentages observed within the study sample and the overall clinic population were analyzed using chi-square statistics, which are appropriate for non-parametric data analysis, using the online GraphPad Prism 9 software (San Diego, CA, USA). Percentages were rounded to the nearest whole number and input for the study sample (observed) and overall clinic population (expected). The chi-square statistic (X^2^), degrees of freedom (d.f.), and appropriate probability (*p*) values were calculated [24,25,26]. In addition, the odds ratio (OR) or odds of occurrence within a particular group, and the relative risk (RR) or risk of risk of occurrence in one group relative to another, were also calculated using a 95% confidence interval by utilizing the online GraphPad Prism 9 software (San Diego, CA, USA).

## 3. Results

A total of N = 122 study samples were identified within the biorepository for inclusion in the current study (Table 1). The analysis of the available samples revealed that the study samples were nearly equally divided between males (*n* = 65/122 or 53.2%) and females (*n* = 57/122 or 46.7%), which was not significantly different from the overall clinic patient population from which the samples were collected, *p* = 0.6891. Analysis of the racial and ethnic distribution within the study sample revealed that the majority of samples were originally derived from minority (non-White) patients (*n* = 78/122 or 63.9%), which was similar to the distribution within the overall clinic population, *p* = 0.8339. Further evaluation revealed that the percentage of study samples from Hispanic or Latino patients (*n* = 41/122 or 33.6%) was lower than the proportion of Hispanic or Latino patients within the patient clinic (52.4%), while the percentage of samples from Black or African American patients (*n* = 21/122 or 17.2%) was higher than observed within the clinic population (12.2%). The distribution of Asians or Pacific Islanders was similar between the study sample (*n* = 5/122 or 4.1%) and the overall clinic population (3.8%).

Analysis of the average age of the patient samples included in the study revealed an age of 25.08 years (range 7 to 69 years), which was not significantly different from the average age of 25.67 years seen in the patients of the combined adult and pediatric patient clinics (range 0 to 89 years), *p* = 0.778. However, more detailed analysis revealed that the average age of pediatric study samples (14.09 years) was older than the overall pediatric clinic population (9.04 years), while the average age of adult study samples (33.51 years) was younger than the overall adult clinic population (42.3 years). This was likely due to the more restricted age range of the pediatric study samples (7 to 17 years) due to the need for pediatric assent, which excluded patients younger than seven years, but did not affect the overall pediatric population (0 to 17 years). In addition, the age range of the adult study samples (18 to 69 years) was more restricted than the overall adult clinic population (18 to 89 years), although this was likely due to random selection, voluntary self-selection, and enrollment in the original sample collection and study participation.

To compare the study samples in the current study, one additional variable, body mass index or BMI, was evaluated (Table 2). These data reveal that less than one-quarter of the study sample participants (22.9%) had a BMI within the normal range, which was similar to the percentage of patients within the overall clinic (25.9%) who had a healthy BMI range of BMI 18–24. The percentage of study sample patients with a BMI in the overweight category (BMI 25–29) was also similar to that observed in the overall clinical population (21.3%, 25.4%, respectively). Finally, the percentage of the study subjects in the obese category (BMI 30 and over) was slightly higher (55.7%) than in the overall clinic population (48.7%), but the difference was not statistically significant, *p* = 0.3627. Overall, the average BMI of all study sample patients combined was 29.8, which was similar to the average BMI of 31.28 in the clinic population.

**Figure 1:**
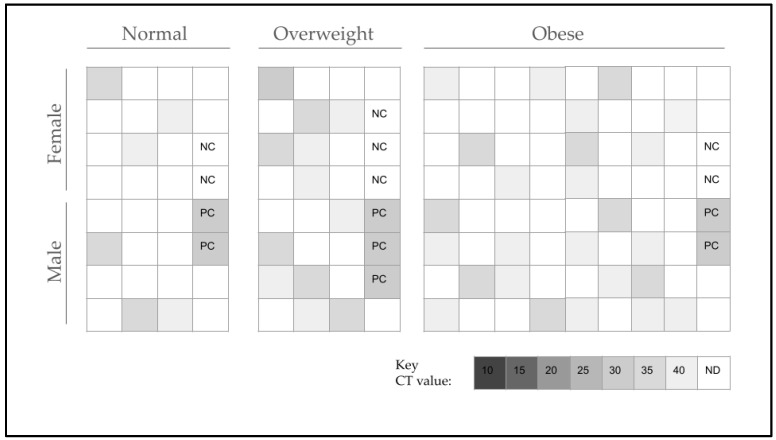
Screening for the presence of *S. noxia* using qPCR. *S. noxia* was found in *n* = 6/28 or 21.4% of samples with a normal BMI (18 to 24), *n* = 12/26 or 46.1% of samples with an overweight BMI (25 to 29), and *n* = 25/68 or 36.8% in the obese BMI (30 and above) category. In total, *n* = 43/122 or 35.2% of study samples tested positive. NC = negative control, PC = positive control, CT = qPCR cycle threshold.

The study samples (*n* = 122) were screened for the presence of *S. noxia* using qPCR and validated primers (Figure 1). The data reveal that *S. noxia* was present in *n* = 6/28, or 21.4%, of those identified to have a normal BMI (18 to 24). However, among those in the overweight-BMI category (25 to 29), *S. noxia* was found to be present in *n* = 12/26, or 46.1%, of these samples. Finally, analysis of the obese category patient samples (BMI 30 and higher) found that *n* = 25/68, or 36.8%, were found to harbor *S. noxia*. In total *n* = 43/122, or 35.2%, of the study samples harbored this organism—although the vast majority (*n* = 37/43 or 86%) were predominantly found among the overweight and obese study samples.

To evaluate whether any other variables were associated with the presence of this organism, the qPCR results were analyzed by demographic characteristics (Table 3). These data reveal that there were no significant differences between the percentages of samples testing positive for *S. noxia* and those testing negative among males (55.8% and 48.1%) and females (44.2% and 51.9%) (*p* = 0.1093). The analysis of race and ethnicity data reveals similar percentages of White or non-Minority participants in the *S. noxia*-positive and -negative categories (39.5% and 34.2%, respectively), which was similar to the results observed among the Minority or non-White samples (60.5% and 65.8%, respectively). The small differences observed were not statistically significant, *p* = 0.2945.

The analysis of the overall average age of *S. noxia*-positive (26.01 years) and *S. noxia*-negative (24.12 years) samples revealed no significant differences, *p* = 0.661. However, more detailed analysis revealed that a significantly higher percentage of pediatric patients between the ages of 7 and 17 years were found to harbor *S. noxia* (*n* = 22/43 or 51.2%) than those that tested negative (*n* = 31/79 or 39.2%). Similarly, adults between the ages of 18 and 69 were found to harbor *S. noxia* in percentages almost similar to the pediatric subjects (*n* = 21/43 or 48.8%) but had a higher proportion of those testing negative for this organism (*n* = 48/79 or 60.6%), which was statistically significant, *p* = 0.0139. 

To further evaluate the qPCR screening results, the BMI data from the study subjects were compared to the findings of *S. noxia* in their saliva samples (Table 4). These data clearly indicate that significant differences in prevalence were found between the *S. noxia*-positive and *S. noxia*-negative samples among the different BMI categories. For example, the percentage of *S. noxia*-positive samples among the normal weight samples was 21.4% compared with 78.6% among the negative samples within this category. Although higher percentages of positive samples were found within the overweight category (46.1%), larger percentages of positive samples were also observed within the obese BMI category (36.8%), and these were statistically different than those among the normal BMI category, *p* = 0.0002. In addition, the analysis of *S noxia*-positive and -negative samples within the normal weight category was compared to the results within the overweight and obese categories using odds ratio (OR), which demonstrated an OR = 3.67, or an increased odds of an *S. noxia*-positive result in the higher BMI categories that was statistically significant, *p* = 0.005895. Finally, the calculation of relative risk (RR) of an *S. noxia*-positive result between the normal weight and overweight or obese categories was performed, revealing a relative risk (RR) of RR = 2.33, which was also statistically significant, *p* = 0.0258.

## 4. Discussion

The primary objective of this study was to analyze saliva samples from an existing biorepository to determine the prevalence of *S. noxia* among this group of clinic patients and to evaluate any demographic or biometric associations in both children and adults. Several dozen samples (N = 122) were successfully identified and screened, revealing the presence of this organism in approximately one-third (35.2%) of samples. Although few studies have examined prevalence, these data are similar to those observed in other recent studies of *S. noxia,* which showed *S. noxia* prevalence in the saliva samples of between 11.1% and 34.4% among pediatric patients to approximately 26.3% among adults [24,25,26]. Most notably, this may be the first study to demonstrate that the prevalence of this organism is most closely associated with BMI rather than other demographic variables, such as age or sex.

This information increases our understanding of oral pathogens and their prevalence, as most of the focus on the development and progress of this organism has been restricted to studies involving other periodontal pathogens, such as *Fusobacterium nucleatum, Porphyromonas gingivalis*, *Tannerella forsythia*, and *Treponema denticola* [27,28]. Moreover, existing studies have outlined the well-documented associations between advancing age and the development of periodontal disease, which do not appear to be similar to the observations from this study and the distribution of *Selenomonas* within the study sample [29,30]. Although strong evidence demonstrates that the development of gingivitis and periodontal disease may be more common among adolescents and young adults undergoing orthodontic treatment, none of the subjects included in this study had fixed appliances or braces [31,32,33]. In addition, *S. noxia* was found in similar percentages in both pediatric and adult samples, which may suggest that the prevalence of this organism is related to factors other than those of the more traditional periodontal pathogens discussed above [34,35].

Another significant finding from this study was the lack of association between the sex of each subject and the distribution of *Selenomonas* within the study samples. Many other studies have found significant differences between males and females with respect to the development and progression of periodontal disease and the prevalence of specific periodontal pathogens [36,37,38]. The lack of sex-specific associations with this organism is consistent with other recent studies that have included both pediatric and adult patient populations, strongly suggesting that the traditional risk factors of sex and age may not be the most important factors to determine which patients harbor *Selenomonas* [24,25].

The clear association between microbial prevalence in saliva samples from overweight and obese patients strongly suggests that some interplay between the dietary and behavioral factors that influence BMI may be responsible for the observations revealed by this study [39,40]. In fact, a growing body of evidence suggests that overweight and obesity may have significant links with the development and progression of periodontal disease, particularly among children and adolescents [41,42,43]. However, the association between *Selenomonas* and periodontal disease, combined with the evidence suggesting that this organism is also associated with overweight and obese patients without periodontal disease, may suggest that more complex and inter-related pathways are involved [22,23,44,45]. 

Despite the overall significance of these findings, there are also limitations associated with this study that should also be considered. For instance, this study relied on the screening of an existing saliva biorepository and may therefore have been influenced by temporal factors, such as dietary habits and BMI prevalence when the sample was taken (e.g., 2012 versus 2022) [46,47]. In addition, due to the retrospective nature of this study, no other systemic or health information that might have been useful to contextualize these results was available for these subjects [48,49,50]. Future studies may involve the prospective collection of this type of additional data, such as neck circumference, exercise data, or dietary patterns, which may be useful in determining whether other factors are associated with the acquisition and colonization of the oral cavity by this organism [51]. In addition, screening for other oral microbial constituents may reveal additional patterns of co-existence or inhibition and complex interactions that may yield significant information regarding the epidemiology of this unusual oral pathogen.

These results suggest that *Selenomonas* may play an important and significant role in a two-way system that influences the development of overweight and obesity, although this may not be the only periodontal pathogen with specific properties that influence this relationship. For example, other studies have demonstrated that additional periodontal pathogens, including *Aggregatibacter actinomycetemcomitans*, *Treponema denticola,* and *Tannerella forsythia,* may also be observed to have different prevalence between normal weight and overweight or obese patients with periodontal disease [52,53]. However, the bidirectional relationships observed between these pathogens and systemic diseases, such as diabetes, metabolic syndrome, and obesity, appear to be more functionally related to inflammatory cytokines and the inhibition of other metabolic pathways rather than on directly increasing caloric recovery from dietary intake, as has been demonstrated with *Selenomonas* [54,55]. These results clearly demonstrate that more research is needed to determine the connections and relationships that exist between overweight and obesity and the presence of this (and other) periodontal pathogen(s).

## 5. Conclusions

These data strongly suggest that the prevalence of oral *S. noxia* may be intricately associated with overweight and obesity among dental clinic patients, a pattern which was observed to be similar in both children and adults. Moreover, the prevalence of this organism appeared to be influenced not only by traditional biometric and demographic factors. such as sex and age, although orthodontic status has yet to be evaluated. This divergence from the established patterns warrants further investigation. Future research studies are needed in this area to determine the positive and negative feedback mechanisms that may be responsible for these observations. Additionally, understanding the specific types of dental or other oral healthcare interventions that could limit or reduce this oral pathogen could have significant implications for public health. This study facilitates new avenues for exploring the complex interplay between oral microbiota, obesity, and overall health.

## Figures and Tables

**Table 1:** Demographic analysis of study samples.

Demographic	Study Sample (*n* = 122)	UNLV-SDM Clinic Population	Statistical Analysis
*Sex*			
Male	53.2% *n* = 65/122	50.9%	X^2^ = 0.160, d.f. = 1 *p* = 0.6891
Female	46.7% *n* = 57/122	49.1%	
*Race or Ethnicity*			
White/non-minority	36.1% *n* = 44/122	34.6%	X^2^ = 0.044, d.f. = 1 *p* = 0.8339
Minority/non-White	63.9% *n* = 78/122	65.4%	
Hispanic/Latino	33.6% *n* = 41/122	52.4%	
Black/Afr. Amer.	17.2% *n* = 21/122	12.2%	
Asian/Pac Islander	4.1% *n* = 5/122	3.8%	
Other	7.4% *n* = 9/122	3.0%	
Age			
Average	25.08 years 14.09 years (Pediatric) 33.51 years (Adult)	25.67 years 9.04 years (Pediatric) 42.3 years (Adult)	Two tailed *t*-test *p* = 0.778
Range	7–17 years (Peds) 18–69 years (Adult)	0–17 years (Peds) 18–89 years (Adult)	

**Table 2:** Analysis of study sample and clinic population body mass index (BMI).

Demographic	Study Sample (*n* = 122)	UNLV-SDM Clinic Population	Statistical Analysis
*Body Mass Index*			
Normal BMI 18–24	22.9% *n* = 28/122	25.9%	X^2^ = 2.028, d.f. = 2 *p* = 0.3627
Overweight BMI 25–29	21.3% *n* = 26/122	25.4%	
Obese BMI 30 and over	55.7% *n* = 68/94	48.7%	
Average (Overweight/Obese)	29.8 overall 32.51 above normal	31.28 overall	

**Table 3:** Demographic analysis of qPCR screening results.

Demographic	*S. noxia* Positive	*S noxia* Negative	Statistical Analysis
*Sex*			
Males	55.8% (*n* = 24/43)	48.1% (*n* = 38/79)	X^2^ = 2.564, d.f. = 1 *p* = 0.1093
Females	44.2% (*n* = 19/43)	51.9% (*n* = 41/79)	
*Race/Ethnicity*			
White/Caucasian	39.5% (*n* = 17/43)	34.2% (*n* = 27/79)	X^2^ = 1.099, d.f. = 1 *p*= 0.2945
non-White/Minority	60.5% (*n* = 26/43)	65.8% (*n* = 52/79)	
*Age*			
Average	26.01 years	24.12 years	Two-tailed *t*-test *p* = 0.661
Age 7–17	51.2% (*n* = 22/43)	39.2% (*n* = 31/79)	X^2^ = 6.053, d.f. = 1 *p* = 0.0139
Age 18–69	48.8% (*n* = 21/43)	60.6% (*n* = 48/79)	

**Table 4:** BMI analysis of qPCR screening results.

Demographic	*S. noxia* Positive	*S. noxia* Negative	Statistical Analysis
*BMI*			
Normal weight BMI 18–24	21.42% (*n* = 6/28)	78.57% (*n* = 22/28)	X^2^ = 13.619, d.f. = 2 *p* = 0.0002
Overweight BMI 25–29	46.1% (*n* = 12/26)	53.9% (*n* = 14/26)	OR = 3.67 95% CI [1.33, 10.08] *p* = 0.005895
Obese BMI 30–over	36.8% (*n* = 25/68)	63.2% (*n* = 43/68)	RR = 2.33 95% CI [1.108, 4.91} *p* = 0.0258

OR (odds ratio), RR (relative risk), CI (confidence interval).

## Data Availability

Due to the protocol approval specifications, any additional data from this study may only be made available directly from the study authors.
